# Comparative Proteome and Phosphoproteome Analyses Reveal Different Molecular Mechanism Between Stone Planting Under the Forest and Greenhouse Planting of *Dendrobium huoshanense*

**DOI:** 10.3389/fpls.2022.937392

**Published:** 2022-07-07

**Authors:** Liping Wu, Xiaoxi Meng, Huizhen Huang, Yingying Liu, Weimin Jiang, Xinglong Su, Zhaojian Wang, Fei Meng, Longhai Wang, Daiyin Peng, Shihai Xing

**Affiliations:** ^1^College of Pharmacy, Anhui University of Chinese Medicine, Hefei, China; ^2^Department of Horticultural Science, University of Minnesota, St. Paul, MN, United States; ^3^Hunan Key Laboratory for Conservation and Utilization of Biological Resources in the Nanyue Mountainous Region, College of Life Sciences and Environment, Hengyang Normal University, Hengyang, China; ^4^College of Humanities and International Education Exchange, Anhui University of Chinese Medicine, Hefei, China; ^5^School of Integrated Chinese and Western Medicine, Anhui University of Chinese Medicine, Hefei, China; ^6^Institute of Traditional Chinese Medicine Resources Protection and Development, Anhui Academy of Chinese Medicine, Hefei, China; ^7^MOE-Anhui Joint Collaborative Innovation Center for Quality Improvement of Anhui Genuine Chinese Medicinal Materials, Hefei, China; ^8^Anhui Province Key Laboratory of Research and Development of Chinese Medicine, Hefei, China

**Keywords:** proteome, phosphoproteome, stone planting under the forest, greenhouse planting, *Dendrobium huoshanense*

## Abstract

The highly esteemed Chinese herb, *Dendrobium huoshanense*, whose major metabolites are polysaccharides and alkaloids, is on the verge of extinction. The stone planting under the forest (SPUF) and greenhouse planting (GP) of *D. huoshanense* are two different cultivation methods of pharmaceutical Dendrobium with significantly differences in morphology, metabolites content and composition, and medication efficacy. Here, we conducted proteomics and phosphoproteomics analyses to reveal differences in molecular mechanisms between SPUF and GP. We identified 237 differentially expressed proteins (DEPs) between the two proteomes, and 291 modification sites belonging to 215 phosphoproteins with a phosphorylation level significantly changed (PLSC) were observed. GO, KEGG pathway, protein domain, and cluster analyses revealed that these DEPs were mainly localized in the chloroplast; involved in processes such as posttranslational modification, carbohydrate transport and metabolism, and secondary metabolite biosynthesis; and enriched in pathways mainly including linoleic acid metabolism, plant-pathogen interactions, and phenylpropanoid, cutin, suberin, and wax biosynthesis. PLSC phosphoproteins were mainly located in the chloroplast, and highly enriched in responses to different stresses and signal transduction mechanisms through protein kinase and phosphotransferase activities. Significant differences between SPUF and GP were observed by mapping the DEPs and phosphorylated proteins to photosynthesis and polysaccharide and alkaloid biosynthesis pathways. Phosphorylation characteristics and kinase categories in *D. huoshanense* were also clarified in this study. We analyzed different molecular mechanisms between SPUF and GP at proteomic and phosphoproteomic levels, providing valuable information for the development and utilization of *D. huoshanense*.

## Introduction

**FIGURE 1:**
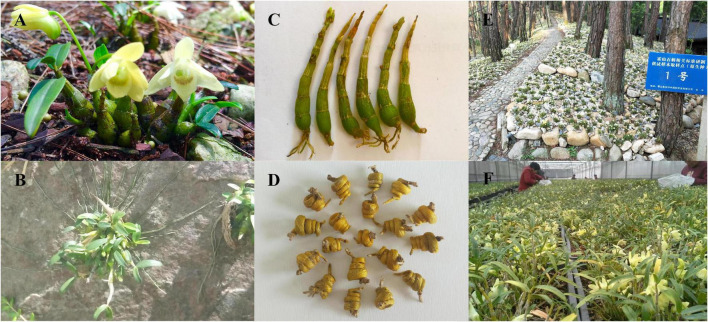
Figures of the of *Dendrobium huoshanense* original plant, its habitat, medicinal part, processing products, and cultivating pattern. **(A)** The original plant of *D. huoshanense*. **(B)** *D. huoshanense* can live on the stone, indicating the derivation of its Chinese name. **(C)** The stem is the medicinal part of *D. huoshanense*. **(D)** “Huoshan Fengdou,” the processed product of *D. huoshanense*. **(E)** Stone planting under the forest (SPUF). **(F)** Greenhouse planting (GP).

*Dendrobium huoshanense* C.Z. Tang et S.J. Cheng (Huoshan Shi-hu, 霍山石斛, “石” means stone) (Figure 1A), commonly known as “Mihu,” is a perennial, aerial, and epiphytic herb that grows on stone (Figure 1B) and belongs to the family Orchidaceae (Wang et al., 2019). It is commonly used as a valuable high-end medicinal herb and is only found in the northern mountain regions of the Yangtze River in China. The *D. huoshanense* stem has medicinal properties (Figure 1C) and fresh stems are often processed into “Huoshan Fengdou” (Figure 1D; Niu et al., 2018), the main commodity in markets. The annual production of *D. huoshanense* exceeded 40 tons in 2020 in China with a total output value of 2 billion Chinese Yuan (Chen and Xia, 2020). Increasing studies have shown that *D. huoshanense* possesses considerable pharmacological activities, involved in cataract prevention (Ge et al., 2018), immunomodulation, tumor proliferation inhibition, decreases in blood lipid and glucose levels (Wang et al., 2014), and hepatoprotection (Tian et al., 2015). Moreover, *D. huoshanense* has been used as a functional food ingredient to produce tea drinks, porridges, and soups, *D. huoshanense* comprises medicinal, nutritional, and ornamental functions and has great prospects for further development (Hao et al., 2018).

The active medicinal components of *D. huoshanense* are very complex, including polysaccharides (Deng et al., 2016), alkaloids (Liang et al., 2019), flavonoids (Chang et al., 2010), and other compounds. Polysaccharide is one of the most important active components of medicinal plants and its pharmacological activities were influenced greatly by its content and monosaccharide compositions (Yuan et al., 2018). Therefore, the content of total polysaccharide and main monosaccharides are the essential quality evaluation standards of plant medicines, especially for plants with polysaccharides as the main active components, such as *D. huoshanense* (Tian et al., 2013). Studies have shown that the major monosaccharide components of polysaccharides in *D. huoshanense* and *Dendrobium officinale* are glucose and mannose (Xing et al., 2015; Hao et al., 2018). The mannose-containing polysaccharides obtained from natural plants exhibit higher bioactivities than those of glucose-containing polysaccharides and other monosaccharides (Yu et al., 2018). Studies have shown that alkaloids are also important bioactive compounds in *D. huoshanense* (Jin et al., 2016).

The wild *D. huoshanense* is often epiphytic to rocks and tree trunks under forest at an altitude of 200–1,200 m (Yuan et al., 2018; Chen et al., 2020), can grow well on wet stone walls facing northwest and northeast or cliff stones with waterfalls, and often forms small communities with mosses and lichens (Chen et al., 2020). Due to the unique growth environment, slow growth rates, and excessive manual collection, the wild resources of *D. huoshanense* are seriously threatened and cannot meet market demand (Jin et al., 2016). Thus, the stone planting under the forest (SPUF) and greenhouse planting (GP) of *D. huoshanense* have already become its major pharmaceutical source. SPUF restored the wild growth environment of *Dendrobium* to a certain extent, broad-leaved forest, coniferous forest or coniferous broad-leaved mixed forest with good ventilation, moderate density and thick trunk in the hillside were selected, and the canopy closure is 0.4–0.6, stones and moss are used as substrates (Figure 1E). GP was evolved by controlling the temperature (room temperature), humidity (80%), ventilation in a greenhouse. The substrate is a mixture of stone and bark in a certain proportion. In summer, a sunshade is used to prevent direct sunlight. In winter, a plastic film is installed for thermal insulation. Spray and ventilation and cooling facilities are set inside to control the external environment to make it suitable for the growth of *Dendrobium* (Figure 1F). The two cultivation modes have different environmental conditions including light, temperature, soil conditions, pH, humidity, etc., which consequently affect the accumulation of primary and secondary metabolites, and ultimately lead to a different chemical composition and efficacy (Yang et al., 2018). Studies had established the characteristic chromatogram of flavonoids in wild and cultivated *D. huoshanense* by High performance liquid chromatography (HPLC) characteristic chromatogram analysis technology, and found that there was difference in flavonoid content under different cultivation modes (Wei et al., 2014). The results also showed significant differences in the content of polysaccharides and ethanol extracts under different cultivation patterns (Chen et al., 2015). Moreover, long-term exposure to a harsh environment might contribute to the accumulation of metabolites, which can elevate the quality of traditional Chinese medicine (Bowne et al., 2012; Brunetti et al., 2013). A similar study has shown that *Dendrobium moniliforme* (L.) Sw. can alleviate drought stress by changing its physiological mechanism. Additionally, the primary and secondary metabolites of *D. moniliforme* were improved at the stage of antagonism with the environment (Wu et al., 2016). However, the molecular mechanism of the differences in *D. huoshanense* composition under different planting modes, such as SPUF and GP, has not been reported, especially at proteomic and phosphoproteomic level. Based on the differences in morphology, physiology and metabolites, especially the content of polysaccharides and alkaloids, and composition of polysaccharides, we inferred there were significant discrepancy on protein abundances and protein post-translational modification levels between GP and SPUF of *D. huoshanense*.

Protein phosphorylation/dephosphorylation, catalyzed by kinases and phosphatases, was first discovered (Levene and Alsberg, 1996). This post-translational modification (PTM) has become one of the most widely studied reversible protein modifications. It is involved in the regulation of many cellular functions through activation/inhibition of enzymes and transporters, functional protein localization, and signal transduction (Ghelis, 2011). Studies have shown that protein phosphorylation is involved in the signal recognition and transduction of plants under abiotic stress. For example, tyrosine phosphorylation of Arabidopsis RACK1A (Receptor for Activated C Kinase 1A) protein regulates multiple environmental stress signaling pathways, including drought and salt stress resistance pathways (Sabila et al., 2016). The classical mitogen-activated protein kinase (MAPK) signaling pathway is activated through phytohormones, reactive oxygen species (ROS), and calcium ions, and the phosphorylation process transfers the upstream signal to downstream response molecules, finally inducing the stress response (Zhao et al., 2017). In eukaryotes, phosphorylation mostly occurs on hydroxylated amino acid residues such as threonine (Thr), serine (Ser), and tyrosine (Tyr) (Pearlman et al., 2011).

In this study, comparative proteome and phosphoproteome analyses were performed to quantitatively display the protein abundance and phosphorylation status between SPUF and GP, and attempt to reveal the mechanisms for the different accumulation of metabolites and biological adaptability between them.

## Materials and Methods

### Plant Materials, Morphology Measurements, and Reagents

The greenhouse planting (GP) and stone planting under the forest (SPUF) materials were collected from *D. huoshanense* cultivated on a hill under a forest of Taipingfan town Huoshan County, Anhui, China and whose man-made environment imitated a wild-type one, and three independent 3-year-old whole plants had been collected for the samples. All samples used for proteome and transcriptome analysis between SPUF and GP were whole plants with the same batch cultivated in the same year. The morphology index such as stem length and stem diameter were measured with vernier caliper.

The TMT 10plex™ Isobaric Mass Tag Labeling Kit, trypsin, and BCA Protein Assay Kit were purchased from ThermoFisher Scientific, Promega Corporation, and Beyotime Biotechnology, respectively. Acetonitrile was obtained from Fisher Chemical, and other chemicals [trifluoroacetic acid, formic acid, iodoacetamide, dithiothreitol (DTT), urea, trichloroacetic acid, protease inhibitor, EDTA, and TEAB] were all purchased from Sigma-Aldrich. Standards of (+)-glucose, Dendrobine were purchased from Chengdu Push Bio-technology Co., Ltd., Chengdu, China.

### Extraction and Determination of Water-Soluble Polysaccharides and Alkaloids

Polysaccharides in *D. huoshanense* were extracted, and polysaccharides content was determined by the phenol-sulfuric acid method described by Chinese Pharmacopoeia (National Pharmacopoeia Committee, 2020). The polysaccharides content was calculated using glucose as a standard. There were three biological replicates for each sample in this experiment.

Bush et al.’s method was modified to analyze alkaloid contents (Bush et al., 1997; Yuan et al., 2019). The total alkaloid content was calculated using Dendrobine as a standard. There were three biological replicates for each sample in this experiment.

### Polysaccharide Hydrolysis and Monosaccharide Determination

Dried *D. huoshanense* powder (0.12 g) was defatted with 80% alcohol at 80°C for 4 h and then extracted with 100 mL of distilled water at 100°C for 1 h, as previously described (Mohan et al., 2020). The filtered supernatant (1.0 mL) was hydrolyzed with 0.5 mL of 3.0 M HCl at 110°C for 1 h in a sealed glass and neutralized to pH 7.0 with 3.0 M NaOH after being cooled to room temperature. The hydrolyzed products (0.4 mL) were added to a solution of 1-phenyl-3-methyl-5-pyrazolone (PMP) consisting of 0.3 M aqueous NaOH (0.4 mL) and 0.5 M methanol (0.4 mL), and then kept at 70°C for 100 min in an oven. After neutralization with 0.3 M HCl, the PMP derivatives were determined in an Agilent Series 1260 system (Agilent Technologies, Shanghai, China) equipped with a Topsil C18 HPLC column (4.6 mm × 250 mm, 5 μm particle size) and monitored by UV absorbance at 250 nm. Elution was carried out using (A) 0.02 M amine acetate solution and (B) acetonitrile as a mobile phase. The ratio of A to B was 80:20. The flow rate was 1.0 mL/min, the sample injection volume was 10 μL, and the column temperature was 30°C. Glucose (100 μg/ml) and mannose (100 μg/ml) were used as internal standards, and there were three biological replicates for each sample in this experiment were used.

### Proteome Analysis

#### Protein Extraction and Western Blot Analysis

Proteins were extracted according to the procedures of previous study (Dong et al., 2021), and separated using 15.0% SDS-PAGE gel and electro-transferred to a PVDF membrane (Millipore) for Western blots. After blocking for 1 h with 5% skimmed milk in Tris-buffered saline with Tween^®^ (TBST), the membrane was incubated overnight at 4°C in a 1/1000 dilution of rabbit-derived pan anti-phosphotyrosine antibody (Thermo Pierce). The unbound primary antibody was removed by washing thrice with TBST buffer (10 min each). Then, the membrane was incubated in a 1/10000 dilution of Goat Anti-Mouse IgG (H + L), Peroxidase Conjugate (Thermo Pierce) at 25°C. The unbound secondary antibody was also removed by washing thrice with TBST buffer (10 min per wash). Enhanced chemiluminescence reagent (LABLEAD, Beijing, China) was used for the detection of proteins in the Western blot.

#### Trypsin Digestion of Proteins

For protein digestion, proteins extracted from all samples in three duplicates were reduced with 5 mM dithiothreitol for 30 min at 56°C, and then were alkylated with 11 mM iodoacetamide for 15 min at room temperature in the dark. Then, 100 mM TEAB (triethylammonium bicarbonate) was added to dilute the urea concentration to less than 2 M. Trypsin in sequencing grade was added in a 1:50 (trypsin: protein, w/w) ratio for the first digestion overnight. Finally, a second 4-h digestion with trypsin was performed at a 1:100 (w/w) ratio for complete protein digestion.

#### Tandem Mass Tag Labeling of Peptides

Peptides were desalted with a Strata X C18 SPE column (Phenomenex), dried under vacuum, and re-dissolved with 0.5 M TEAB following the manufacturer’s protocol for the TMT labeling kit. The specific steps were as follows: after thawing, the labeled reagent was dissolved in acetonitrile, mixed with the peptide and incubated at room temperature for 2 h. After mixing, the labeled peptide was desalted and vacuum freeze-dried.

#### High Performance Liquid Chromatography Fractionation of Peptides

High pH reverse-phase HPLC fractionation of the peptide samples after digestion was performed with an Agilent 300 Extend C18 column (5-μm particles, 10 mm ID, 250 mm length). In short, a gradient of 8% to 32% acetonitrile (pH 9.0) over 60 min was used to fractionate the sample into 60 fractions. Then, peptides were further combined into 18 fractions and dry-centrifuged under vacuum.

### LC-MS/MS Analysis

Formic acid (0.1%), as solvent A, was used to desalt the peptides, while a reversed-phase analytical column (15 cm length, 75 μm inner diameter) was used to load the peptides instantly. The concentration of solvent B (0.1% formic acid in 98% acetonitrile) in the gradient increased from 6 to 23% for 26 min, increased from 23 to 35% for 8 min, and increased to 80% in 3 min, and then reached 80%, being held for the last 3 min. All steps were performed on the EASY-nLC 1000 UPLC system at a constant flow rate of 400 nL/min. Peptides were processed with an NSI source and then subjected to tandem mass spectrometry (MS/MS) analysis in Q ExactiveTM Plus (ThermoFisher Scientific, Guangzhou, China) connected to UPLC. The applied electrospray voltage was 2.0 kV, and the m/z scan range of the full MS scan was 350–1800. The integral peptides were observed at a resolution of 70,000 in Orbitrap, and the peptides were selected as MS/MS using standardized collision energy, the number was set to 28, and the ion fragment was detected resolution at 17,500 in Orbitrap. A data related process alternates between 1 MS scan and 20 MS/MS scans, and the dynamic exclusion was 15.0 s. Automatic gain control (AGC) was set to 5E4. The first mass setting was kept at 100 m/Z.

### Database Search

We used the Maxquant search engine (v.1.5.2.8) to handle the obtained MS/MS data. Tandem mass spectra of the data were searched against a transcriptome database uploaded by us,^1^ combined with a reverse decoy database. Trypsin/P was consigned as the cleavage enzyme, and there were up to 2 missing cleavages; the minimum length of the peptide segment was set to 7 amino acid residues. For precursor ions, the mass error was set to 20 ppm in the first search and 5 ppm in the main search, followed by 0.02 Da for ion fragments. The quantitative method was set to TMT-6plex, and the FDR of protein identification and PSM (peptide-spectrum matches) identification were both set to 1%.

### Bioinformatics Analysis

Gene Ontology (GO) annotations of phosphorylated proteins were compared against the UniProt-GOA database.^2^ By GO annotation, proteins are divided into three categories: biological process, cellular compartment, and molecular function.

Pathways associated with the phosphorylated proteins were annotated based on the Kyoto Encyclopedia of Genes and Genomes (KEGG) database. Wolfpsort software was applied to predict the subcellular localization of the phosphorylated proteins in *D. huoshanense.*

For GO, KEGG pathway, and protein domain enrichment analyses, a two-tailed Fisher’s exact test was used to examine the differentially expressed proteins and phosphorylated proteins against all identified proteins. Multiple testing corrections were performed utilizing standard false discovery rate control methods, and the terms with a corrected *p*-value < 0.05 were considered significantly enriched. For further hierarchical clustering, we first collated all the categories and *p*-values. *P*-values were log10 (*p*-value) transformed and then z-transformed for each category (*P* < 0.05). The one-way hierarchical clustering method was utilized to cluster z scores. Cluster membership was visualized by heat map utilizing the “heatmap.2” function from the “gplots” R-package.

### Subcellular Localization

Prediction of subcellular localization of proteins by using WoLF PSORT (v.0.25).

### Phosphorylation Protein Analysis

The method of protein extraction, western blot analysis and trypsin digestion was the same as that described in proteome analysis.

### Immobilized Metal Affinity Chromatography Enrichment for Phosphorylation

Firstly, separated peptides were incubated with vibrating Ti-IMAC (immobilized metal affinity chromatography) microsphere suspensions in a loading buffer containing 50% acetonitrile and 6% trifluoroacetic acid. Then, we collected IMAC microspheres enriched in phosphopeptides and discarded the supernatant after centrifugation. Next, aiming at removing non-specific adsorbed peptides, IMAC microspheres were sequentially rinsed with 50% acetonitrile, 6% trifluoroacetic acid, 30% acetonitrile, and 0.1% trifluoroacetic acid. Enriched phosphopeptides were vibrationally diluted with an elution buffer containing 10% NH_4_OH, and collected and lyophilized for LC-MS/MS analysis.

### LC-MS/MS Analysis

Solvent A and Solvent B, the analytical column used in UPLC and the instrument of tandem mass spectrometry (MS/MS) are the same as above. The concentration of solvent B in the gradient increased from 4 to 23% for 40 min, increased from 23 to 35% for 12 min, and increased to 80% in 4 min, and then reached 80%, being held for the last 4 min. The constant flow rate of 300 nL/min was used in UPLC system.

For MS analysis, the m/z scan range of the full MS scan was 350–1600, the integral peptides were observed at a resolution of 60,000, and the ion fragment was detected resolution at 15,000 in Orbitrap. AGC was set to 1E5, and the first mass setting was kept at 100 m/Z.

### Database Search

Cysteine alkylation was set as fixed modification and variable modification as oxidation of methionine, acetylation of protein N-terminus, and phosphorylation of serine, threonine, and tyrosine. The minimum score for modified or non-modified peptides was set to > 40. All of the other steps and parameters are the same as shown in proteome analysis.

### Gene Ontology, Kyoto Encyclopedia of Genes and Genomes Annotation, and Enrichment Analysis

For Gene Ontology, KEGG annotation, and enrichment analysis, the method was also same as that described in proteome analysis.

### Motif Analysis

MoMo Modification Motifs (version5.3.3^3^) (Bailey et al., 2009) software was applied to analyze the conserved amino acid sequence motifs. The Motif-x algorithm (Chou and Schwartz, 2011)^4^ was used to analyze the model of conserved motifs of phosphoryl-13-mers (six amino acids upstream and downstream of the phosphorylation site). All the database protein sequences were used as the background database, other parameters were set to default. Only when the number of peptides of a specific sequence is more than 20 and the *p*-value ≤ 0.000001, the specific amino acid sequence was considered as a conserved characteristic motif.

### Kinase and Phosphatase Classification

According to the downloaded genome release pathway (Zm_B73_5b_FGS_cds_2012), kinases and phosphatases were identified by MapMan software and categorized referring to the maize databases of ProFITS, P3DB,^5^ and ITAK.^6^ We used the P3DB database to perform BLAST searches on the kinase sequences of Arabidopsis and rice.

GPS5.0 software^7^ (Wang et al., 2020) was used for predicting the activity sites of upstream phosphorylation kinases. There is a hypothesis that similar short peptides may act as alike roles and similar protein kinases may regulate alike short-term motifs. Thus, software predictions may affect the kinase protein families by regulating specific phosphorylation sites, then gains the kinases by comparing with iEKPD database (Guo et al., 2019). At last, the upstream kinase family corresponding to the phosphorylation sites and the kinase IDs corresponding to the kinase family in the database were obtained.

### Total RNA Extraction, Construction of cDNA Libraries, and RNA-Seq

The ethanol precipitation protocol and CTAB-pBIOZOL reagent were used to purify total RNA from the whole plants of SPUF and GP according to the manufacturer’s instructions (Bioflux), and each sample was prepared with three biological replicates. RNA concentration and quality were measured using a Nano Drop and Agilent 2100 bioanalyzer (Thermo Fisher Scientific, Waltham, MA, United States). After the RNA sample was qualified, fragment buffer was added to the enriched RNA to break the RNA into small fragments. Then, the first strand of cDNA was synthesized by reverse transcription with 6 bp random primers, and then the second strand was synthesized by adding buffer, dNTPs, DNA polymerase I and RNase H. Finally, cDNA library construction was performed by high-throughput sequencing using the Illumina HiSeq platforms at Novogene.

### Real-Time Fluorescent Quantitative PCR Analysis

RT-qPCR analysis was performed using a SuperReal PreMix Plus SyBr Green PCR kit [Tiangen Biotech (Beijing) Co., Ltd., Beijing, China] on a Cobas z480 Real-Time PCR System, following the method previously described (Wang et al., 2021). Candidate primers were designed using Primer 5.0 (Supplementary Table 1). Reactions contained 2.0 μL of diluted cDNA, 0.6 μL of each primer, 10 μL of 2 × SuperReal PreMix Plus, and 6.8 μL of RNase-free double-distilled water (ddH_2_O). All RT-qPCRs were performed as follows: denaturation at 95°C for 15 min, followed by 45 cycles of 95°C for 10 s, 58°C for 20 s, and 72°C for 30 s. Each sample contained three biological replicates, a housekeeping gene (*18S rRNA*) was used as a reference, and the relative expression level of each gene was calculated using the 2^–ΔΔCt^ approach.

### Statistical Analysis

At least three independent biological replicates were used for physiological and biochemical analyses. The unpaired two-tailed student’s *t*-test was used to determine statistically significant *p*-values in the mean. A significance level of *p*-value < 0.05 and *p*-value < 0.01 were considered as statistically difference and significant difference, respectively. For protein abundance and phosphorylation level analysis, the relative quantitative values were log2 transformed to make the data conform to the normal distribution, and then a two-sample two-tailed *T*-test method was performed. When *p*-value < 0.05 and protein abundance/modification ratio > FC (fold change) was regarded as up-regulation/hyperphosphorylated. When *p*-value < 0.05 and protein ratio < FC was regarded as down-regulation/hypophosphorylated.

## Results

### Differences in Morphology, Polysaccharides, and Alkaloids Between Stone Planting Under the Forest and Greenhouse Planting

**FIGURE 2:**
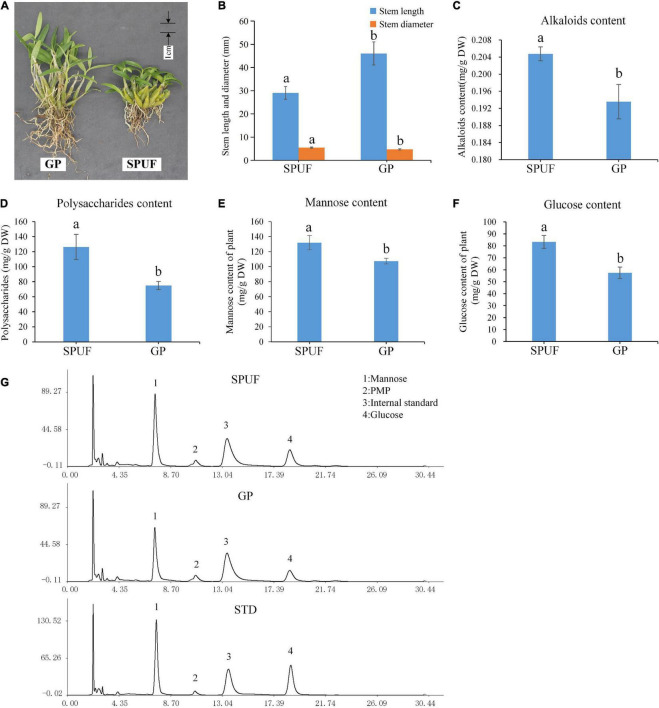
Differences in phenotype and main metabolites content between SPUF and GP. A significance level of *p*-value < 0.05 was considered as statistically significant. **(A)** Phenotype of SPUF and GP. **(B)** Stem length and diameter of SPUF and GP. **(C–F)** Alkaloid, polysaccharide, mannose, and glucose content in SPUF and GP. **(G)** Chromatogram of the main monosaccharide composition of SPUF and GP in *D. huoshanense* derived by PMP (1-phenyl-3-methyl-5-pyrazolone). Glucosamine hydrochloride was used as internal standard. Different letters above bars indicate significant differences (*P* < 0.01).

Significant differences in the morphology index and main metabolites content of SPUF and GP were revealed (Figure 2A). The stem length of GP was much higher than that of SPUF, while SPUF had a larger stem diameter than that of GP (Figures 2A,B and Supplementary Table 2). The leaf to total plant mass ratio in GP was higher than that in SPUF. However, the reverse relationship was found for the root to total plant mass ratio (Supplementary Table 2). Total alkaloids and total polysaccharides content in SPUF were higher than those in GP (Figures 2C,D and Supplementary Table 3). Moreover, regarding polysaccharide composition, mannose and glucose levels in SPUF were higher than those in GP (Figures 2E,F and Supplementary Table 3). These results indicated that the cultivation modes of SPUF was more conducive to the accumulation of active constituents of polysaccharide and alkaloid.

### Global Proteome and Phosphoproteome Profiling in *Dendrobium huoshanense*

**FIGURE 3:**
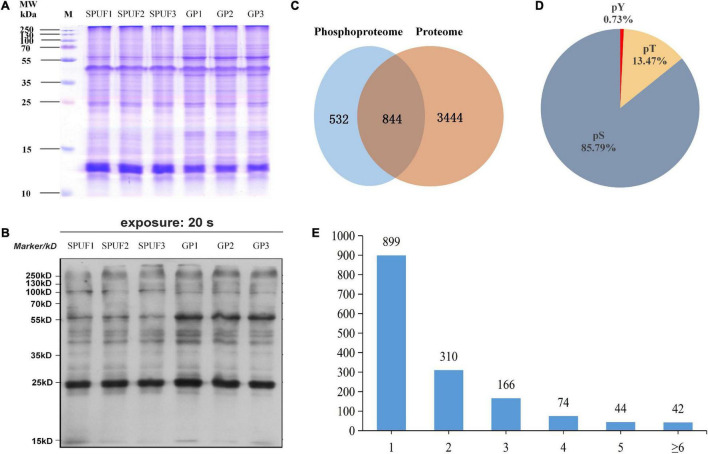
Estimation and determination of global protein phosphorylation site occupancy in *D. huoshanense*. **(A)** SDS-PAGE image of total protein stained with Coomassie Brilliant Blue G-250. Molecular weight is labeled in the left, samples and their duplicates are labeled on the top. M, marker; SPUF1, SPUF2, and SPUF3 are duplicates of stone planting under the forest; GP1, GP2, and GP3 are duplicates of the greenhouse planting. **(B)** Western blot image of protein phosphorylation. The primary antibodies used were rabbit-derived pan anti-phosphotyrosine antibodies (Jingjie PTM Biolab, Hangzhou, China). **(C)** Venn diagram of proteins identified between the proteome and phosphoproteome analysis. **(D)** Frequency of identified pS, pT, pY phosphosites. **(E)** Distribution of phosphopeptides. More than half of the phosphorylated sites were confidently singly phosphorylated.

Aiming at establishing a comprehensive database specifically for the proteome and phosphoproteome of *D. huoshanense*, we extracted the proteins from SPUF and GP in three independent biological replicates (Figure 3A). A western blot experiment was performed to verify the phosphorylation of proteins in SPUF and GP by Pan anti-phosphotrysine antibody (Figure 3B). As a result, different patterns were revealed in proteome and phosphoproteome by SDS-PAGE and Western blot, indicating different protein composition and protein phosphorylation status between SPUF and GP (Figures 3A,B). Subsequently, the digested peptides of proteome and enriched peptides of phosphoproteome were identified by liquid chromatography followed by mass spectrometry (LC/MS) analysis. The mass spectrometry proteomics data of this study have been deposited into the ProteomeXchange Consortium *via* the PRIDE partner repository with the dataset identifier PXD022337.

A total of 4,820 *D. huoshanense* proteins were identified in this study by proteome and phosphoproteome analysis (Figure 3C and Supplementary Table 4). Among them, 3,504 proteins for which quantitative information was available were identified in a comparative proteomics study (Figure 3C), and 1,376 proteins containing 2,113 phosphorylated sites were obtained by screening based on localization probability ≥ 0.75 in a phosphoproteomic study (Figure 3C and Supplementary Table 4). Regardless of the localization information, a total of 1,535 proteins containing 2,872 phosphorylated sites were identified (Supplementary Table 5).

Approximately 85.79% of the detected phosphorylated sites were pSer (pS), 13.47% pThr (pT), and 0.73% pTyr (pY) (Figure 3D), more than half of the phosphopeptides were singly phosphorylated (Figure 3E). There were 42 proteins with more than six phosphorylation sites, and the one with the highest degree of phosphorylation was annotated as “PREDICTED: RNA-binding motif protein, X chromosome-like isoform X2 [Phoenix dactylifera]” and contained 12 pS (Supplementary Table 4). Among these identified proteins, a total of 1,400 proteins with 2,329 phosphorylated sites were quantifiable (Supplementary Table 5).

### Characterization of Serine-, Threonine-, and Tyrosine-Phosphorylated Peptides

**FIGURE 4:**
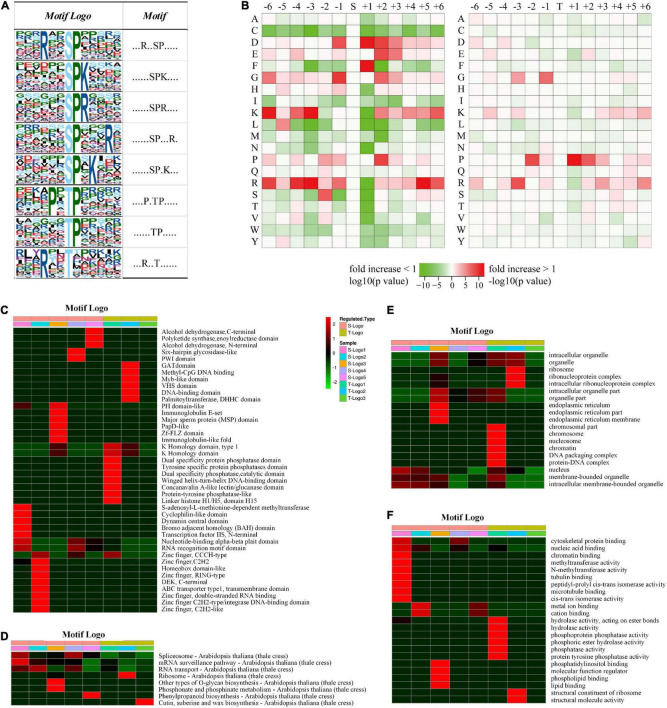
Motif and logo-based clustering analysis of the phosphorylation sites. **(A)** Eight representative conserved motifs of phosphoryl-21-mers flanking phosphorylation sites (“S” and “T,” which represent serine and threonine, respectively, are at position 0). The size of each letter correlates to the frequency of that amino acid residue occurring in that position. **(B)** Heat map of the amino acid compositions around the phosphorylation sites. The –log10 (Fisher’s exact test *p*-value) for every amino acid in each position (from –6 to +6) is shown, phosphorylation sites in serine are shown on the left, and phosphorylation sites in threonine on the right. Motif logo-based clustering analyses: **(C)** protein domain enrichment analysis, **(D)** KEGG pathway enrichment analysis, **(E)** biological process enrichment analysis, and **(F)** molecular function analysis.

To understand the regulation and amino acid residue preference around serine, threonine, and tyrosine phosphorylation sites, motif analysis was performed for the sequence from sites −6 to +6 of all the 2,329 phosphorylation sites. From the 1,673 conserved peptides, 24 conserved motifs were identified, of which 21 contained serine residues, 3 contained threonine residues, and none contained tyrosine residues (Supplementary Table 6). Eight distinguished motifs were identified (marked as Motif Logo): 5 conserved serine motifs (with a high value fold increase) such as SPK, SPR, RXXSP, SPXK, SPXXXR (S indicates the phosphorylated serine, R, P, K indicate arginine, proline, and lysine, respectively, and X indicates a random amino acid residue) and 3 conserved threonine motifs such as PXTP, TP, and RXXT (T indicates the phosphorylated threonine), as shown in Figure 4A. Additionally, the frequencies of amino acid residues flanking the phosphorylated serine and threonine were analyzed to demonstrate the enrichment or depletion of various amino acids (Figure 4B and Supplementary Table 7).

It is intriguing that a significant preference for motifs was discovered in phosphorylated proteins associated with different domains, pathways, biological processes, and cellular components (Figures 4C–F). For example, peptides involved in biosynthetic and metabolic processes preferentially harbor the motif TphP (Figure 4C). Endoplasmic reticulum-related cellular components preferentially harbor the motif SphPR, and chromosome-related cellular components preferentially harbor the motif PXTphP (Figure 4E). Hydrolase- and phosphatase-related molecular functions preferentially harbor the motif PXTphP (Figure 4F). The identification of conserved motifs and their preferences in different protein clusters verified that, instead of a non-specific modification, protein phosphorylation is a highly regulated modification in *D. huoshanense*, whose function is affected by the composition of neighboring amino acid residues.

### Differentially Expressed Proteome Analysis

Differentially expressed proteomes between GP and SPUF were profiled by a tandem mass tag (TMT)-labeled LC-MS quantitative proteome method with three biological replicates. Among the identified proteins, 3,504 proteins containing quantitative information were identified in the comparative proteomic study (Figure 3C and Supplementary Table 4). A total of 611 proteins were revealed to be differentially expressed between SPUF and GP with a fold change (FC) ≥ 1.5 (upregulated) or ≤ 0.667 (downregulated) and *p*-value ≤ 0.05. Among these 611 differentially expressed proteins (DEPs), 310 proteins were upregulated and 301 were downregulated in GP compared to their levels in SPUF. The protein with the highest FC value was predicted as a NUCLEAR FUSION DEFECTIVE 4-like protein, with a FC of 26.477 (*p*-value: 2.19E-6) (Supplementary Table 8).

### Functional Annotation of Differentially Expressed Proteins

To thoroughly understand the function and characteristics of the DEPs, subcellular localization, functional classification, functional enrichment, cluster of orthologous groups, and protein domain enrichment analyses of these proteins were performed.

Subcellular localization analysis of these DEPs (Supplementary Table 9) revealed that most (54.6%) were in chloroplasts, including 279 proteins (176 upregulated and 103 downregulated in GP vs. SPUF), indicating that photosynthesis is significantly different between SPUF and GP. The second and third largest classes for DEPs were in the cytoplasm and nucleus (Supplementary Table 9).

Gene ontology (GO) analysis of the 611 DEPs suggested that metabolic and cellular process, as well as proteins with catalytic and binding activity, were significantly divergent between SPUF and GP (Supplementary Table 10).

**FIGURE 5:**
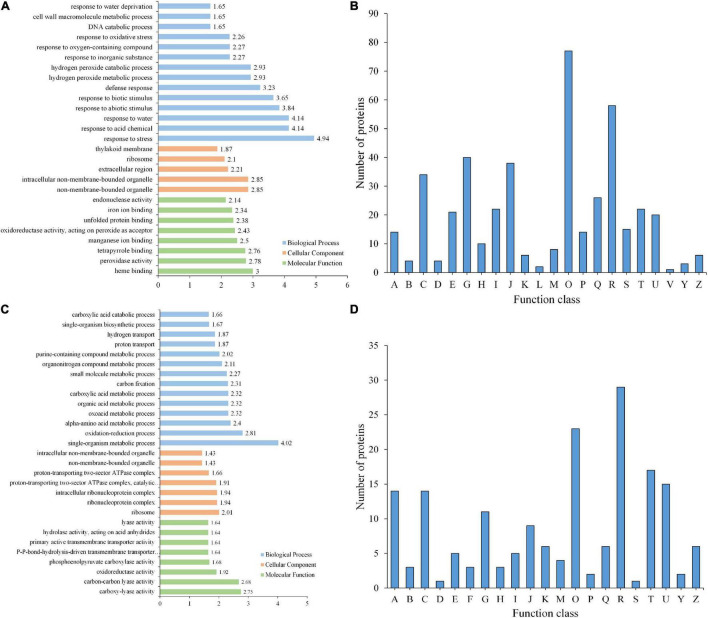
GO enrichment and KOG functional analyses of differentially expressed proteins. **(A)** GO enrichment analysis of DEPs. **(B)** KOG analysis of DEPs. **(C)** GO enrichment analysis of PLSC phosphoproteins. **(D)** KOG analysis of PLSC phosphoproteins. A, RNA processing and modification; B, Chromatin structure and dynamics; C, Energy production and conversion; D, Cell cycle control, cell division, chromosome partitioning; E, Amino acid transport and metabolism; F, Nucleotide transport and metabolism; G, Carbohydrate transport and metabolism; H, Coenzyme transport and metabolism; I, Lipid transport and metabolism; J, Translation, ribosomal structure and biogenesis; K, Transcription; L, Replication, recombination, and repair; M, Cell wall/membrane/envelope biogenesis; O, Posttranslational modification, protein turnover, chaperones; P, Inorganic ion transport and metabolism; R, General function prediction only; S, Function unknown; T, Signal transduction mechanisms; U, Intracellular trafficking, secretion, and vesicular transport; Z, Cytoskeleton.

From GO enrichment analysis, these DEPs were mainly enriched in biological processes such as responses to biotic and abiotic processes (GO:0009628 and GO:0009607) and other defense processes (GO:0006950, GO:0001101, GO:0009415, and GO:0006952) (Figure 5A and Supplementary Table 11). Among these DEPs, proteins responding to abiotic stimuli were enriched with the highest −log10 (*p*-value), both upregulated and downregulated DEPs between GP and SPUF. In GP, most of those DEPs were enriched in responses to water (GO:0009415), heat (GO:0009408), acid (GO:0001101), and inorganic substances (GO:0010035), as well as enriched in biosynthesis (GO:0072525, GO:0009108, GO:0051188, and GO:0044085). Proteins pertaining to ion homeostasis (GO:0055082, GO:0048878, GO:0006879, GO:0055076, and GO:0055072) and catabolic processes (GO:0042744, GO:0006308, and GO:1901136) were enriched in SPUF (Supplementary Table 11). These enrichments of DEPs are better explained by differences in growth conditions and morphology between SPUF and GP. Many enriched proteins involved in biotic and abiotic stress and ion homeostasis grant SPUF an increased tolerance to harsh habitat.

Based on KOG/COG (cluster of orthologous groups) functional analysis, SPUF and GP proteins showed differences in posttranslational modifications, protein turnover, chaperones, and carbohydrate transport and metabolism (Figure 5B). Additionally, many DEPs participated in the biosynthesis, transport, and catabolism of secondary metabolites, as well as energy production and conversion (Figure 5B). KOG/COG analysis showed that DEPs upregulated in SPUF mainly belonged to pathways such as plant-pathogen interactions, alpha-linolenic acid metabolism, and linoleic acid metabolism, phenylpropanoid biosynthesis (Supplementary Figure 1A), which were mainly localized in the nucleus, cytoskeleton, and non-membrane-bounded organelles (Supplementary Figure 1B). Proteins with a higher expression in GP were mainly categorized into pathways associated with amino acid metabolism, carbon fixation in photosynthesis, carbon metabolism, zeatin biosynthesis, etc. (Supplementary Figure 1A), which were mainly localized in thylakoids and the photosynthetic membrane (Supplementary Figure 1B).

Based on the Kyoto Encyclopedia of Genes and Genomes (KEGG) pathway enrichment analysis, proteins in GP were enriched in pathways such as glyoxylate and dicarboxylate metabolism; glycine, serine, and threonine metabolism; carbon metabolism; zeatin biosynthesis; carbon fixation in photosynthetic organisms; flavone and flavonol biosynthesis; tropane, piperidine, and pyridine alkaloid biosynthesis; and thiamine metabolism. Proteins abundant in SPUF were involved in pathways such as linoleic acid metabolism; plant-pathogen interactions; fatty acid elongation; ribosome biosynthesis; alpha-linolenic acid metabolism; phenylpropanoid biosynthesis; cutin, suberin, wax, and unsaturated fatty acid biosynthesis (Supplementary Table 12).

Protein domain enrichment analysis showed that proteins with aquaporin-like, bet v I/Major latex, START (star-related lipid-transfer)-like, secretory peroxidase, PLAT/LH2 (lipase/lipooxigenase domain), and heat shock protein Hsp90 (N terminal) domains were enriched in SPUF compared to their enrichment in GP (Supplementary Table 13), which indicated that these proteins may increase the resistance of SPUF to water, heat, oxidation, and other stresses.

### Differential Phosphorylation Analysis

Phosphoproteome analysis between GP and SPUF was carried out by phosphorylation affinity enrichment technology and TMT-labeled high-resolution LC-MS. Among the identified phosphoproteins, a total of 1,400 with 2,329 phosphorylated sites were quantifiable (Supplementary Table 5). The phosphorylation level significantly changed (PLSC) on 291 modified sites belonging to 215 phosphoproteins with a FC ≥ 1.5 (upregulated) or ≤ 0.667 (downregulated) and *p*-value ≤ 0.05 based on three biological replicates (Supplementary Table 14). Among them, 56 hypophosphorylated and 235 hyperphosphorylated sites were detected in GP compared to their levels in SPUF.

### Functional Annotation of Phosphorylation Level Significantly Changed Phosphoproteins

The 215 PLSC phosphoproteins containing 291 phosphorylated modification sites underwent GO annotation and GO enrichment analyses. Distribution bar charts of biological process (BP), cellular component (CC), and molecular function (MF) are shown in Figure 5C and Supplementary Table 15. From the BP perspective, single-organism metabolic process (GO:0044710), oxidation-reduction process (GO:0055114), alpha-amino acid metabolic process (GO:1901605), oxoacid metabolic process (GO:0043436), etc., were significantly enriched. In terms of CC, ribosome (GO:0005840), ribonucleoprotein complex (GO:1990904), intracellular ribonucleoprotein complex (GO:0030529), proton-transporting two-sector ATPase complex, catalytic domain (GO:0033178), etc., were highly enriched. Regarding MF, carboxy-lyase activity (GO:0016831), carbon-carbon lyase activity (GO:0016830), oxidoreductase activity, acting on the aldehyde or oxo group of donors, NAD or NADP as acceptor (GO:0016620), phosphoenolpyruvate carboxylase activity (GO:0008964), etc., were significantly enriched relative to the background (Figure 5C and Supplementary Table 16).

Kyoto Encyclopedia of Genes and Genomes enrichment analysis of PLSC proteins in SPUF revealed that the pathways of glyoxylate and dicarboxylate metabolism, endocytosis, and oxidative phosphorylation were enriched. The pathways of pyruvate metabolism, taurine and hypotaurine metabolism, histidine metabolism, and glycerolipid metabolism were enriched in GP. The pathway of beta-alanine metabolism was enriched in both GP and SPUF (Supplementary Table 17). The pathways with PLSC phosphorylated protein enrichment in SPUF were closely associated to plant resistance and secondary metabolism, while those in GP were related to plant growth and development. These results reasonably account for the higher metabolite content and better adaptation to worse growth conditions of SPUF despite the larger biomass of GP.

KOG analysis suggested that a substantial number of PLSC phosphoproteins were involved in post-translational modification, signal transduction, and different carbohydrate transport and metabolism for adaptation to different growth environments (Figure 5D). Differential transport and metabolism of carbohydrates results in different contents of carbohydrate-associated metabolites between GP and SPUF.

Moreover, subcellular localization analysis suggested that PLSC phosphoproteins are largely located in chloroplasts, with 72 (33%) proteins (57 hyperphosphorylated and 15 hypophosphorylated in GP vs. SPUF), followed by the nucleus and cytoplasm (Supplementary Table 18). Since several PLSC phosphoproteins located in chloroplast are involved in metabolic processes and have catalytic and binding activity, phosphorylation likely plays a role in photosynthesis regulation in SPUF and GP and may result in different morphology traits and carbohydrate accumulation.

These results implied that these PLSC proteins were highly enriched in response to different stresses and signal transduction mechanism processes in GP and SPUF and caused different carbohydrate transport and metabolism, which might associate with differences in polysaccharide content and composition.

### Protein Kinases and Phosphatases in *Dendrobium huoshanense*

We further exploited our extensive phosphoproteomics dataset and predicted the potential enzymes responsible for phosphorylation. Since there are no related phosphatase databases for the phosphatase analysis of *D. huoshanense* at present, only kinases were analyzed in this study. Altogether, 677 phosphosites catalyzed by kinases were predicted from the 2,329 identified phosphosites. These kinases catalyzing phosphoproteins were assigned to eight categories involving CAMK, TKL, CMGC histone kinase, STE, Atypical, AGC, CK1, and others in *D. huoshanense* (Supplementary Table 19). The percentages of phosphosites in *D. huoshanense* catalyzed by major kinases such as CAMK, TKL, and CMGC were 15.84, 26.63, and 28.65%, respectively (Supplementary Table 19). From bioinformatics analysis and prediction, 911 putative kinases in *D. huoshanense* may be involved in catalyzing these phosphosites. For instance, ATPase family AAA domain-containing protein 1-A isoform X3 is a kinase that might be involved in the modification of 5 phosphosites, and its expression level in GP is 1.71 times higher than that in SPUF (Supplementary Table 20).

### Validation by Real-Time Fluorescent Quantitative PCR

**FIGURE 6:**
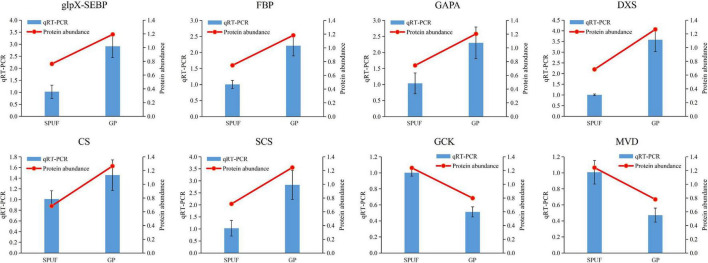
qRT-PCR analysis of genes encoding enzymes differentially expressed in major metabolites biosynthesis. The *18S rRNA* was used as an internal reference gene for normalization. Blue bars represent qRT-PCR data and red lines indicate protein abundance from proteomic analyses. Data represent mean ± standard error of three replicates. The left Y-axis denotes relative expression levels of genes determined by qRT-PCR, and the right Y-axis denotes protein abundance data.

We verified the transcript level of relevant genes which encode enzymes involved in the Calvin cycle and polysaccharide and alkaloid biosynthesis pathways, such as glpX-SEBP, FBP, GAPA, DXS, CS, SCS, GCK, and MVD. Transcriptional levels of glpX-SEBP, FBP, GAPA, DXS, CS, and SCS in GP were higher than those in SPUF. Meanwhile, the expression levels of GCK and MVD in SPUF were higher than those in GP (Figure 6). These qRT-PCR results were consistent with data from the proteome analysis.

## Discussion

### SPUF *Dendrobium huoshanense* Contains Higher Active Constituents

Different cultivation modes lead to great changes in traits, quality, and effects. To reveal differences between SPUF and GP, we conducted a systematic comparison of their appearance and quality. The stems of SPUF are short and thick, resembling the wild *D. huoshanense*. Our study found that the content of major secondary metabolites in SPUF is significantly higher than that in GP (Figure 2), which is consistent with previous reports (Yi et al., 2021). Since mannose-containing polysaccharides exhibit higher bioactivities than those of glucose-containing polysaccharides and other monosaccharides (Yu et al., 2018), it is conceivable that SPUF, with a higher content of mannose and polysaccharides, is more medicinally effective than GP. It had been reported that the protective effect against mouse liver injury of SPUF was better than that of GP (Li et al., 2020). And the study had verified that SPUF was more similar to wild *D. huoshanense* and the quality of its medicinal materials was considerably better than that of GP’s medicinal materials (Yi et al., 2021). The fresh and dried stem of SPUF tastes bitterer, which might be due to its higher content of alkaloids compared to GP. From the literature and our results, SPUF is more suitable for medicine than GP.

### Protein Phosphorylation in *Dendrobium huoshanense*

Various cellular activities are controlled by protein PTMs, and more than 200 types of PTMs have been identified, such as phosphorylation, methylation, acetylation, and ubiquitination (Krishna and Wold, 1993). Protein kinase cascades play essential roles in diverse intracellular signaling processes in animals and yeast (Tetlow et al., 2004). In plants, phosphorylation is involved in signaling pathways triggered by abiotic stress, pathogen invasion, and plant hormones (Knetsch et al., 1996; Sheen, 1996; Zhang et al., 1998). Additionally, protein phosphorylation is also important in metabolism, transcription, translation, proteolysis, homeostasis, and signaling (Thingholm et al., 2009). Despite the importance and widespread occurrence of protein phosphorylation in plants, this is the first report to date on the proteome and phosphoproteome profile of *D. huoshanense* to the best of our knowledge. In this study, a total of 532 specific proteins were identified in the phosphoproteome by an immobilized metal affinity chromatography (IMAC) sphere phosphopeptide enrichment approach, which were not identified in the proteome (Figure 3C). Protein phosphorylation is the most abundant and extensively studied PTM (Ptacek and Snyder, 2006), with approximately 30% of proteins being phosphorylated (Hauser et al., 2017). Here, the percentage of protein phosphorylation was 28.55% (Figure 3C). The occurrence of identified phosphosites were pSer > pThr > pTyr in our study (Figure 3D), which was consistent with most plants (Rattanakan et al., 2016; Chen et al., 2017). The less frequent nature of tyrosine phosphorylation in plants might due to their lack of receptor tyrosine kinases (Luan, 2002). Studies have shown that mono-phosphosites in phosphoproteins are dominant (Rattanakan et al., 2016; Chen et al., 2017). In our study, the percentage of mono-phosphorylated peptides was 58.57% (Figure 3E). The above results demonstrate the reliability of this study’s results.

### Analysis of Differentially Expressed Kinases and Phosphorylation Level Significantly Changed Kinases

Significant differences in conditions such as temperature, light, water, soil composition, soil pathogens, pH (Figures 1E,F) may affect the accumulation of metabolites in plants and ultimately lead to different chemical compositions and efficacies between SPUF and GP. These environmental conditions influence signal transduction, and the widespread nature of phosphorylation in plants renders it an ideal model to explore the molecular basis of differences between SPUF and GP. There were 19 differentially expressed kinases and 12 PLSC kinases identified in the proteomics and phosphoproteomics studies, respectively. We also found 14 phosphatases differentially expressed and 2 PLSC phosphatases between GP and SPUF, among these DEPs, the levels of 8 were upregulated and 6 were downregulated in GP compared with those in SPUF (Supplementary Table 21). Overall, 9 upregulated and 10 downregulated kinases were differentially expressed in GP. A D-glycerate 3-kinase involved in the reaction of the photorespiratory C2 cycle, an indispensable ancillary metabolic pathway to the photosynthetic C3 cycle (Boldt et al., 2005), was a differentially expressed kinase with the highest fold difference in GP compared with SPUF; while the expression of CBL-interacting serine/threonine-protein kinase 24, a kinase conferring salt tolerance in plants (Yang et al., 2019), in SPUF was much higher than that in GP. The expression of all PLSC kinases generating pSer in GP was higher than that in SPUF, while most of them were located in the chloroplast (Supplementary Table 21). It is suggested that SPUF requires more functional kinases than GP for signal transduction to adapt to harsh environment.

### Difference Expressed and Phosphorylation Level Significantly Changed Enzymes Involved in Photosynthesis

**FIGURE 7:**
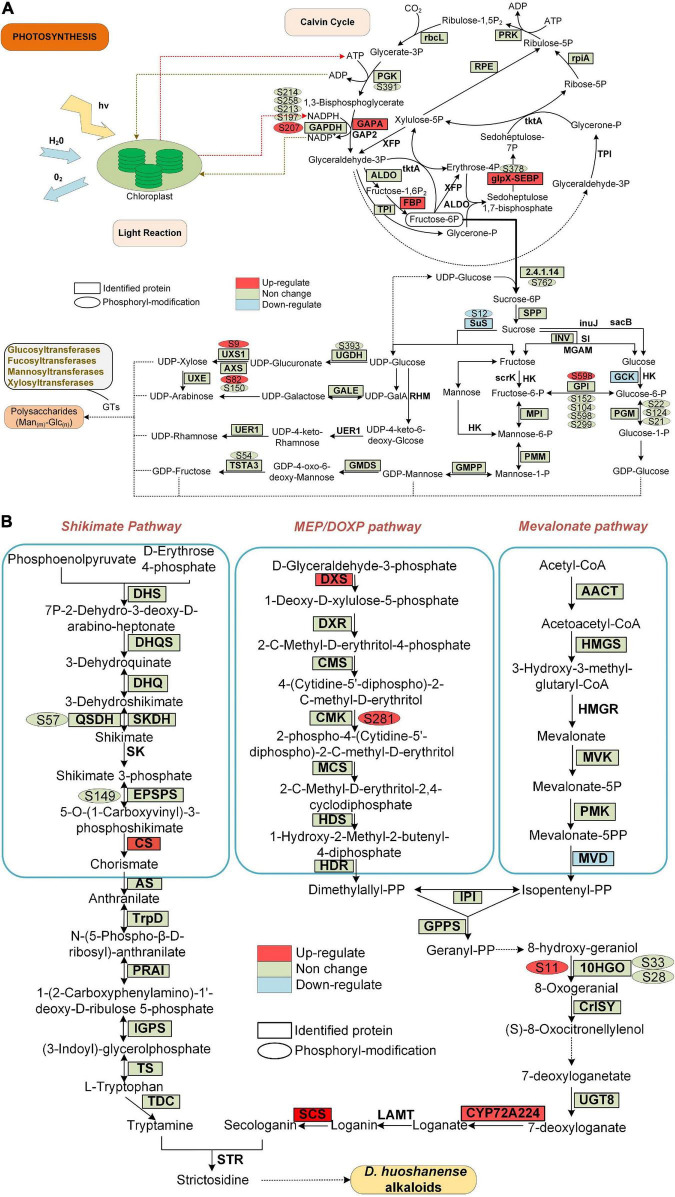
Proteins and phosphoproteins involved in photosynthesis and alkaloid biosynthesis pathways in *D. huoshanense*. Proteins identified in proteome analysis are surrounded by a square frame, and phosphorylated proteins are shown with an oval frame near them. Upregulated and downregulated proteins are shown in GP compared to SPUF. **(A)** Proteins and phosphoproteins involved in photosynthesis. **(B)** Proteins and phosphoproteins involved in alkaloid biosynthesis pathways.

Photosynthesis involves enzymes that are highly heat-sensitive, and previous studies have shown that the phosphorylation of photosynthesis proteins plays an important role in photosynthesis regulation (Boex-Fontvieille et al., 2014; Pan et al., 2018). There were 21 enzymes involved in the photosynthesis pathway identified in this study. The expression of two putative enzymes (FBP: fructose-1,6-bisphosphatase with EC: 3.1.3.11, and petH: ferredoxin-NADP reductase with EC: 1.18.1.2) in GP was higher than that in SPUF, while the PPC candidate enzyme (EC: 4.1.1.31, phosphoenolpyruvate carboxylase 2) had a higher expression in SPUF (Supplementary Table 22). We identified 11 enzymes with 39 phosphorylated sites involved in photosynthesis between SPUF and GP. Glyceraldehyde-3-phosphate dehydrogenase A (GAPA, EC: 1.2.1.13) is an essential enzyme for glycolysis/gluconeogenesis and mediates carbon fixation in photosynthetic organisms. Five sites in GAPA were phosphorylated, among them, Ser120 and Thr121 were PLSC sites (Supplementary Table 22). Glyceraldehyde-3-phosphate dehydrogenase (GAPDH, EC 1.2.1.12) is a key enzyme in the Calvin cycle that catalyzes the reductive dephosphorylation of 1,3-bisphosphoglyceric acid (BPGA) to inorganic phosphate and glyceraladehyde-3-phosphate (Trost et al., 2006; Mekhalfi et al., 2014). Six serine and three threonine sites in GAPDH were phosphorylated in both GP and SPUF (Figure 7A and Supplementary Table 22), but the phosphorylation level of Ser207 changed significantly between them. The phosphorylation of Ser120 and Thr121 of GAPA and Ser207 of GAPDH may be related to the biomass and polysaccharide content of GP and SPUF.

### Differentially Expressed Proteins and Phosphorylation Level Significantly Changed Proteins Involved in Polysaccharides and Alkaloids Biosynthesis

The main bioactive metabolites in *D. huoshanense* are polysaccharides and alkaloids (Hsieh et al., 2008; Matus et al., 2008). Previous studies showed that the whole crotonylation proteome of *D. huoshanense* was analyzed, and the enzyme genes of Calvin cycle, polysaccharide and alkaloid pathway were also crotonylated (Wu et al., 2022). In our study, we found a significant difference in the content of both polysaccharides and alkaloids between GP and SPUF (Figure 2). We found that the protein abundance and phosphorylation level in chloroplasts are significantly different between GP and SPUF. We mapped the proteins identified in our proteome and phosphoproteome analyses to the Calvin cycle and polysaccharide and alkaloid biosynthesis pathways (Figure 7). We found that most of the enzymes involved in these pathways had been identified in our research, and many of them were phosphorylated. We also found that several enzymes were significantly differentially expressed and the phosphorylation level of some phosphorylated sites was significantly changed between GP and SPUF. GAPA, FBP, and glpX-SEBP in the Calvin Cycle (Figure 7A) and CS, DXS, SCS, and CYP72A224 in alkaloid biosynthesis (Figure 7B) were highly expressed in GP. The expression of Sus and GCK, involved in polysaccharide biosynthesis, and MVD, involved in alkaloid biosynthesis, in SPUF was higher than that in GP. NADPH had the most abundant modified sites, with five sites among proteins involved in these pathways, and the phosphorylation level of Ser207 in GP was higher than that in SPUF. Other sites also had higher phosphorylation levels, such as Ser9 of UXS1, Ser82 of AXS, and Ser598 of GP1 in polysaccharides synthesis and Ser281 in CMK and S11 in 10HGO in GP. Only the phosphorylation level of Ser12 of Sus was higher in SPUF.

There is no report on the correlation between protein phosphorylation and polysaccharides or alkaloids biosynthesis. In this study, we found that there may be a correlation between phosphorylation levels of different enzyme sites and the polysaccharide content, including Ser9 of UDP-arabinose 4-epimerase 3 isoform (UXS1), Ser82 of UDP-D-xylose synthase (AXS), Ser598 of glucose-6-phosphate isomerase (GPI), Ser12 of sucrose synthase 1 (Sus) (Figure 7A). There may also be a correlation between the phosphorylation levels and alkaloid content at various enzyme sites, including Ser281 of 4-diphosphocytidyl-2-C-methyl-D-erythritol kinase (CMK) and Ser11 of mannitol dehydrogenase (10HGO) (Figure 7B).

## Conclusion

Altogether, 4,820 proteins were identified by proteome and phosphoproteome analyses in this study from *D. huoshanense*. Among them, 237 were differentially expressed between SPUF and GP, and 215 PLSC phosphoproteins containing 291 modification sites were identified in the phosphoproteome analysis. Gene ontology, KEGG pathway, protein domain, and cluster analyses revealed that these DEPs were mainly localized in the chloroplasts, involved in processes such as posttranslational modification, carbohydrate transport and metabolism, and secondary metabolite biosynthesis, and enriched in pathways mainly including linoleic acid metabolism, plant-pathogen interactions, and phenylpropanoid, cutin, suberin, and wax biosynthesis. PLSC proteins were also mainly located in chloroplasts, and highly enriched in different stress responses and signal transduction mechanisms processes through protein kinase and phosphotransferase activities. These differences accounted for the much stronger tolerance of SPUF to harsh environments, and likely for the obvious differences in morphology between SPUF and GP. Significant differences between SPUF and GP were observed by mapping DEPs and PLSC proteins to photosynthesis and polysaccharide and alkaloid biosynthesis pathways. The phosphorylation characteristics and kinase categories of *D. huoshanense* were also clarified in this study, which constitutes the first proteomic and phosphoproteomic report on *D. huoshanense*. The results might be helpful to understand the mechanism of these two different varieties and improve the levels of cultivation, management and breeding of *D. huoshanense*.

## Data Availability Statement

The proteome data can be found below: PRIDE with ProteomeXchange, PXD022337 (https://www.ebi.ac.uk/pride/archive/projects/PXD022337).

## Author Contributions

SX, LWu, and DP conceived, designed, and implemented the study. LWu and SX collected specimens and prepared samples for sequencing. LWu, XS, DP, ZW, FM, HH, YL, LWa, and WJ statistics analyzed and interpreted. SX, DP, ZW, and YL reagents, materials, and analyzed tools. SX, LWu, and XM drafted the manuscript. All authors edited the manuscript and approved the final version.

## Conflict of Interest

The authors declare that the research was conducted in the absence of any commercial or financial relationships that could be construed as a potential conflict of interest.

## Publisher’s Note

All claims expressed in this article are solely those of the authors and do not necessarily represent those of their affiliated organizations, or those of the publisher, the editors and the reviewers. Any product that may be evaluated in this article, or claim that may be made by its manufacturer, is not guaranteed or endorsed by the publisher.

## Footnotes

1 https://pan.baidu.com/s/1bdA4oE_LjkhC41eePsv0pg. Access code: 2290.

2
http://www.ebi.ac.uk/GOA/

3
https://meme-suite.org/meme/meme_5.3.3/

4
http://motif-x.med.harvard.edu/

5
http://p3db.org/

6
http://bioinfo.bti.cornell.edu/cgi-bin/itak/index.cgi

7
http://gps.biocuckoo.cn/

## Supplementary Material

The raw data and annotated MS spectra of *Dendrobium huoshanese* are available in the proteomics repository PRIDE with ProteomeXchange data set accession number of PXD022337 (for reviewing, the username is reviewer_022337@ebi.ac.uk and password is fHJF6HMM). The transcriptome data generated during the current study as reference genome information are available in the Baidu Cloud repository: https://pan.baidu.com/s/1bdA4oE_LjkhC41eePsv0pg, and access code is 2290.

Supplementary Figure 1 Cluster analysis of the DEPs from proteome.

Supplementary Table 1 Gene descriptions and primers used for qRT-PCR.

Supplementary Table 2 Morphology characteristics of SPUF and GP of *D. huoshanense*.

Supplementary Table 3 Major metabolites and their composition comparison between SPUF and GP of *D. huoshanense*.

Supplementary Table 4 Proteins identified in proteome and phosphoproteome between SPUF and GP of *D. huoshanense*.

Supplementary Table 5 All phosphoproteins were identified without considering the localization probability ≥ 0.75.

Supplementary Table 6 Conserved motif analysis on conserved phosphorylated peptides.

Supplementary Table 7 The −log10 (Fisher’s exact test *p*-value) of the amino acids around the phosphorylation sites in each position (from −6 to +6). Phosphorylation sites in serine are shown on the left and those in threonine are shown on the right.

Supplementary Table 8 Data matrix of differentially expressed proteins from proteome study.

Supplementary Table 9 Subcellular localization categories of the DEPs from proteome of *D. huoshanense*.

Supplementary Table 10 GO classification analysis of the DEPs from proteome of *D. huoshanense*.

Supplementary Table 11 GO enrichment analysis of the DEPs from proteome.

Supplementary Table 12 KEGG pathway enrichment analysis of the DEPs from proteome.

Supplementary Table 13 Proteins domain analysis of the DEPs from proteome.

Supplementary Table 14 Data matrix of phosphorylation level significantly changes proteins from phosphoproteome study.

Supplementary Table 15 GO annotation of the PLSC proteins from phosphoproteome of *D. huoshanense.*

Supplementary Table 16 GO enrichment analysis of the PLSC proteins.

Supplementary Table 17 KEGG pathway enrichment analysis of the PLSC proteins from phosphoproteome of *D. huoshanense.*

Supplementary Table 18 Subcellular localization analysis of the PLSC proteins.

Supplementary Table 19 Kinases predicted in phosphosites identified of *D. huoshanense*.

Supplementary Table 20 Category of the predicted protein kinases of *D. huoshanense.*

Supplementary Table 21 Differentially expressed kinases and phosphatases in proteome and PLSC kinases and phosphatases in phosphoproteome.

Supplementary Table 22 Protein enzymes involved in photosynthesis of *D. huoshanense* identified in proteome and phosphoproteome.

## Abbreviations

SPUF: stone planting under the forest
GP: greenhouse planting
DEPs: differentially expressed proteins
PLSC: phosphorylation level significantly changed
PTM: post-translational modification
RACK1A: receptor for activated C kinase 1A
MAPK: mitogen-activated protein kinase
ROS: reactive oxygen species
Thr: threonine
Ser: serine
Tyr: tyrosine
DTT: dithiothreitol
PMP: 1-phenyl-3-methyl-5-pyrazolone
TBST: tris-buffered saline with Tween^®^
TEAB: triethylammonium bicarbonate
IMAC: immobilized metal affinity chromatography
AGC: automatic gain control
FDR: false discovery rate
GO: gene ontology
KEGG: Kyoto Encyclopedia of Genes and Genomes
FC: fold change
START: star-related lipid-transfer
PLAT/LH2: lipase/lipooxigenase domain
BP: biological process
CC: cellular component
MF: molecular function
RT-qPCR: real-time fluorescent quantitative PCR
FBP: fructose-1,6-bisphosphatase
petH: ferredoxin–NADP reductase
PPC: phosphoenolpyruvate carboxylase
GAPA: glyceraldehyde-3-phosphate dehydrogenase A
GAPDH: glyceraldehyde-3-phosphate dehydrogenase
BPGA: 1,3-bisphosphoglyceric acid
UXS1: UDP-arabinose 4-epimerase 3 isoform
AXS: UDP-D-xylose synthase
GPI: glucose-6-phosphate isomerase
Sus: sucrose synthase
CMK: 4-diphosphocytidyl-2-C-methyl-D-erythritol kinase
10HGO: mannitol dehydrogenase.
