# Supplementary figures and images for: Comparative Proteome and Phosphoproteome Analyses Reveal Different Molecular Mechanism Between Stone Planting Under the Forest and Greenhouse Planting of Dendrobium huoshanense
Source: Front Plant Sci. 2022 Jul 7;13:937392. doi: 10.3389/fpls.2022.937392 (PMC9301318; doi:10.3389/fpls.2022.937392)

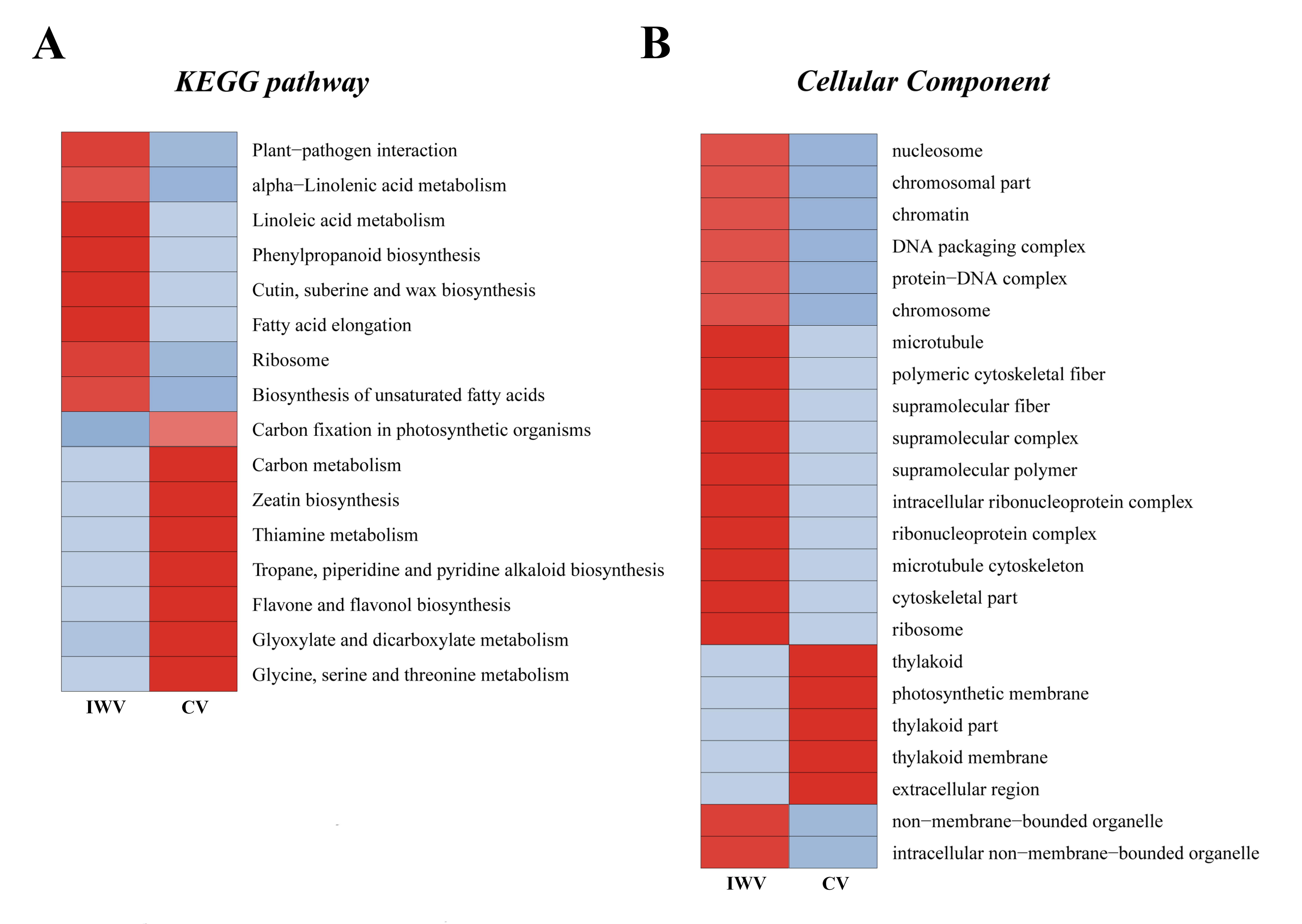

Supplement: Supplementary Figure 1 — Cluster analysis of the DEPs from proteome. [file Data_Sheet_1.ZIP › Supple Tab/Figure S1.jpg]
